# Reinvigoration/Rejuvenation Induced through Micrografting of Tree Species: Signaling through Graft Union

**DOI:** 10.3390/plants10061197

**Published:** 2021-06-11

**Authors:** Isabel Vidoy-Mercado, Isabel Narváez, Elena Palomo-Ríos, Richard E. Litz, Araceli Barceló-Muñoz, Fernando Pliego-Alfaro

**Affiliations:** 1 Fruticultura Subtropical y Mediterránea, IFAPA Centro de Málaga, Unidad Asociada al CSIC, Cortijo de Cruz s/n, 29140 Málaga, Spain; vidoy@uma.es (I.V.-M.); araceli.barcelo@juntadeandalucia.es (A.B.-M.); 2 Departamento de Botánica y Fisiología Vegetal, Facultad de Ciencias, Campus de Teatinos s/n, Instituto de Hortofruticultura Subtropical y Mediterránea “La Mayora” (IHSM-UMA-CSIC), 29071 Málaga, Spain; epalomorios@uma.es (E.P.-R.); ferpliego@uma.es (F.P.-A.); 3 Tropical Research & Education Center, University of Florida, 18905 SW 280 Street, Homestead, FL 33031-3314, USA; relitz@ufl.edu

**Keywords:** reinvigoration, rejuvenation, in vitro grafting, rooting capacity, woody plants, long distance signaling

## Abstract

Trees have a distinctive and generally long juvenile period during which vegetative growth rate is rapid and floral organs do not differentiate. Among trees, the juvenile period can range from 1 year to 15–20 years, although with some forest tree species, it can be longer. Vegetative propagation of trees is usually much easier during the juvenile phase than with mature phase materials. Therefore, reversal of maturity is often necessary in order to obtain materials in which rooting ability has been restored. Micrografting has been developed for trees to address reinvigoration/rejuvenation of elite selections to facilitate vegetative propagation. Generally, shoots obtained after serial grafting have increased rooting competence and develop juvenile traits; in some cases, graft-derived shoots show enhanced in vitro proliferation. Recent advances in graft signaling have shown that several factors, e.g., plant hormones, proteins, and different types of RNA, could be responsible for changes in the scion. The focus of this review includes (1) a discussion of the differences between the juvenile and mature growth phases in trees, (2) successful restoration of juvenile traits through micrografting, and (3) the nature of the different signals passing through the graft union.

## 1. Introduction

The life cycle of trees can be divided into four distinct phases: embryonic, juvenile, transitional, and mature [1]. In the embryonic phase a mature embryo is formed within the seed which can be either orthodox or recalcitrant. The juvenile phase occurs after seed germination and is characterized by an indeterminate growth type due to the proximity of the root system and the reduced plant size [2]. During the transitional phase (vegetative transition), gradual changes in morphology, including growth habit and progressive acquisition of reproductive ability, takes place. Concurrently, juvenile and adult cells are present, and these changes are associated with “ontogenetic maturation” or phase change (transition from juvenile to adult stage in plants) [3]. Reproductive structures can be observed following application of inductive treatments, but plants cannot flower under normal conditions [4,5]. Cuttings or in vitro explants demonstrate a general decline of regenerative ability with increasing age of the mother plant [5]. The mature phase culminates with reproductive maturation, whereas growth rate declines and regenerative ability is progressively lost [5]. Trees require several years to reach maturity, ca. one year for the woody vines *Vitis* spp., 2–8 for *Citrus* and *Prunus* spp., 15–20 for *Acer pseudoplatanus* and *Fraxinus excelsior* and up to 40 years for *Fagus sylvatica* [5]. There occur age-related postmaturation morphological and physiological changes that are linked to increased shoot size resulting in decreased vigor, also known as physiological aging [5]. However, according to Greenwood et al. [2] the developmental decline in height could be due to a change in growth habit with aging rather than the result of a general loss of vigor. Postmaturation changes are not as drastic as in the juvenile–mature transition [6] and can be reversed following successive pruning or grafting onto vigorous rootstocks among other treatments [2,5,7]; afterwards, a reinvigorated (with increased vigor) mother plant is thereby obtained. Similarly, changes related to vegetative maturation are also reversible, although traits do not all behave in a similar manner and do not show the same degree of reversion [8,9]. Reversion to the juvenile stage (rejuvenation) has generally been gauged on the basis of plant morphology rather than by physiological and/or molecular markers. This has made it difficult to differentiate whether reinvigoration (reversion of physiological aging) or rejuvenation (reversion of ontogenetic aging) has been achieved [1,5]. Restoration of a single trait such as rooting ability or a temporary increased vigor, is not an indication that long term rejuvenation, which would include increased growth rate, will also occur [1,5,8]. It is widely accepted that reversion of physiological aging is a prerequisite for rejuvenation [5]. There can also be morphological and physiological changes occurring in shoot appearance due to effects of environmental factors (light, temperature, etc.) which sometimes resemble those occurring in vegetative transition; however, it is not clear whether they share regulatory mechanisms [3].

Differences occur between juvenile and adult materials with respect to hormonal responses [10]; juvenile tissues show a higher hormone sensitivity [6], higher endogenous auxin level [11], or indole-3-acetic acid/abscisic acid (IAA/ABA) ratio [12] while Z-type cytokinins (CKs) have been shown to increase with maturation, showing accumulation at the postflowering stage [13]. Appearance of the J16 membrane-associated protein [14], accumulation of oxygen evolving enhancer protein 2 (OEE2) [15], and appearance of specific restricted fragments of mtDNA [16] have also been found in juvenile in contrast with mature tissues in which a higher level of esterases, peroxidases, tyrosine phosphorilated proteins [17,18], and a higher degree of methylation [6,10,19,20] have been reported.

Investigations with maize have shed light on the role of miRNAs in controlling phase change in plants. Lauter et al. [21] observed that miR172 accumulates during maize shoot development and promotes the transition to the adult phase, being also involved in the degradation of an APETALA2 like transcription factor (Gl15) responsible to maintain juvenile traits. Using maize mutants, Chuck et al. [22] demonstrated that overexpression of miR156 decreased miR172 levels and resulted in maintenance of juvenile traits through overexpression of Gl15. A similar trend occurs in *Arabidopsis* [23]. These observations suggest that relative abundance of these two miRNAs could regulate phase transition in plants [3,22,24]. This has been confirmed in several woody perennials, e.g., *Acacia confusa, Acacia colei, Eucalyptus globulus, Hedera helix, Quercus acutissima*, and *Populus x canadensis* [25], *Malus asiatica* x *Malus domestica* [26], *Persea americana* [27], and *Olea europaea* [28]. With *Macadamia integrifolia* and *Mangifera indica* [27], although abundance of miR156 decreased with age, accumulation of miR172 could not be observed as time progressed. Furthermore, Wang et al. [25] observed a longer juvenile phase in *Populus x canadensis* plants overexpressing miR156, and these authors concluded that this could be the master regulator of juvenility. Moreover, they speculated that it would be desirable to determine if traits of economic importance related with age, e.g., rooting competence, are under the control of miR156. The expression level could also be used to determine the effects of different factors on ontogenetic maturation (phase change) when morphological changes are not associated with vegetative transition. Feng et al. [29], working with tobacco, found several traits, e.g., leaf shape, number of leaf veins, and size and density of epidermal cells, were affected by miR156 expression levels. This marker could be used in the Solanaceae family to better understand changes associated with phase change. With *Prunus* spp. and strawberry, the miR156 expression pattern was altered after in vitro culture, with a marked increase with strawberry and an erratic behavior with *Prunus* spp. [30,31]. Recently Guo et al. [32], working with *Arabidopsis*, showed that miR159 was involved in timing control of phase change through avoidance of continuous activation of miR156. Redox signals and sugars also appear to be involved in modulating miR156 levels [33,34].

With respect to control of morphogenetic capacity, Zhang et al. [35] demonstrated that decreased shoot regeneration competence during aging could be explained by a lower cytokinin sensitivity due to binding of the SQUAMOSA PROMOTER BINDING PROTEIN-LIKE (SPL), a target of miR156, to B-type ARRs, a key factor in cytokinin signaling. In *Malus xiaojinensis* an increase in miR156, ARF7, and ARF9 was observed after 15 in vitro subcultures of adult material with shoots recovering their rooting competence after three additional subcultures [11]. Xu et al. [36] working with juvenile (obtained from suckers of trees of apomictic origin), adult and rejuvenated adult (obtained via meristem culture) leafy cuttings of the same species, demonstrated that lSPL26 was responsible for adventitious root inhibition in adult shoots. Accordingly, targeting of this factor by miR156, found at higher levels in juvenile and rejuvenated adult materials, was responsible for their higher rooting competence. Moreover, miR156 acts independently of PIN and ARF family members in root induction. Heide [37] indicates that rejuvenation observed during adventitious organogenesis and somatic embryogenesis is linked to a shift in expression levels of miR156 and miR172, with accumulation of the former. In *Arabidopsis*, overexpression of miR156 is responsible for enhancing rooting in adult tissues but had no effect on juvenile material [38]; however, these results could not be confirmed by Ye et al. [39], who showed that increasing expression of miR156 in adult leaves of *Arabidopsis thaliana* restored some juvenile traits, i.e., loss of leaf trichomes, but did not increase rooting capacity or cause flowering delay. This could be explained if once the adult phase is reached, epigenetic status of SPL-regulated genes cannot be modified. Ye et al. [39] also suggest that rejuvenation could be associated to changes in DNA sequence, DNA methylation and differential gene expression or that an unknown pathway related with aging occurs and miRNAi induction cannot overcome it. The role of miR156 in rooting capacity has also been questioned, e.g., in *Eucalyptus* spp. in which miR156 was higher in juvenile tissues in comparison with adult material, no relationship between miRNA156 expression and rooting capacity could be found [40]. As previously reported with *Castanea, Hedera, Larix*, and *Pinus* [6], in *Eucalyptus*, decreased rooting competence with age could be explained by several factors, i.e., lower auxin content and sensitivity [41]. Several miRNAs, e.g., miR160, miR167, and miR390, have been shown to play a key role in rooting through modulation of the auxin response [42,43,44,45,46,47]. miRNA169, known to be down regulated by nitrogen starvation [48] is also down regulated in micropropagated strawberry where N deficiency is absent [49] while miRNA390 was upregulated. This controversy prompted Us-Camas et al. [50] to suggest that miRNA regulation could be different in vivo and in vitro, with auxin playing a more pivotal role in vitro, favoring dedifferentiation and enhancing the appearance of juvenile traits.

## 2. Micrografting for Reinvigoration/Rejuvenation of Plus Trees

**Figure 1:**
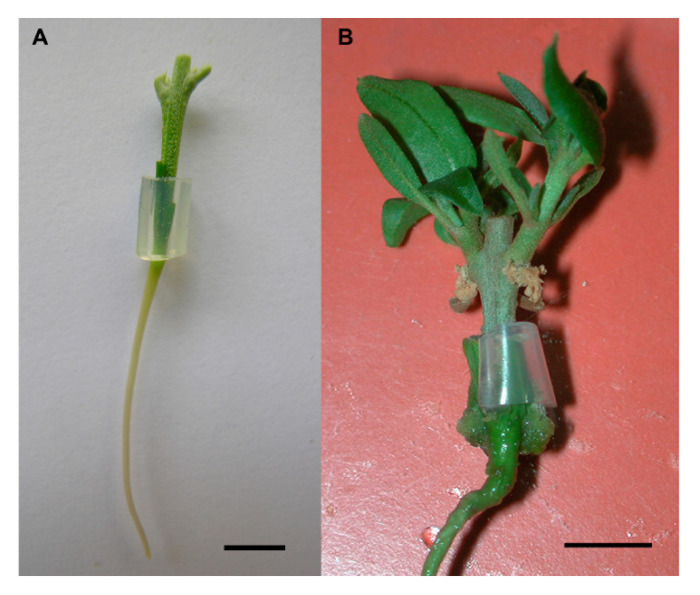
Hypocotyl micrografting in olive. Graft establishment through insertion of nodal section with lateral buds onto the hypocotyl; a silicone ring is used to hold the graft union (**A**). Sprouted shoots after 8 weeks in OM medium supplemented with zeatin (**B**). Bar: 1 cm.

Micrografting involves the in vitro grafting of small shoot apices or lateral buds onto decapitated rootstock seedlings (Figure 1). The efficacy of micrografting as an alternative method for reinvigoration/rejuvenation of ancient trees in forestry and horticulture has been evaluated with different degrees of success. In some cases, partially recovered juvenile morphology in scions and/or restoration of rooting competence or in vitro establishment of rejuvenated material was reported (Table 1) while in others, micrografting resulted in growth enhancement of the scion and no further evaluation of other morphogenic responses (capacity to form roots, axillary or adventitious shoots, changes in leaf morphology) of graft-derived material was indicated (Table 2). 

Reinvigoration/rejuvenation can also be achieved following sequential grafting of mature scions onto juvenile rootstocks in vivo, i.e., in *Cupresuss dupreziana* appearance of acicular juvenile type leaves and increased rooting capacity was observed after a single graft while three grafts were required to observe leaves with juvenile morphology in *Eucaliptus camaldulensis*; in *Pseudotsuga menziesii* use of microscions accelerated the appearance of juvenile traits [7]; *Eucalyptus trabutii, Pinus caribaea, Pinus oocarpa, Quercus acutissima*, and *Tectona grandis* could also be rooted from cuttings, although responses varied with species [104]; there are also reports in which material resulting from in vivo grafts could be successfully established and multiplied in vitro, e.g., *Castanea sativa* [105] and *Pinus massoniana* [12]. However, the possibility of shortening intervals between grafts, the proximity of the scion to the root system, a more precise control of environmental conditions, and the possibility of using a culture medium with hormonal supplements have made in vitro grafting preferable over in vivo grafting for restoration of juvenile traits in adult scions [104,106]. Monteuuis [65] obtained much better results with *Acacia mangium* for graft uptake and scion vigor with in vitro rather than with in vivo grafting. It was suggested that graft miniaturized scion from mature trees used in vitro would have cells close to the apical meristem with more capacity to form callus, facilitating the formation of a successful graft union. Nursery grafting is widely used to rescue adult clones and prepare material for other rejuvenation treatments [104]. In *Pistacia vera* grafted onto *Pistacia terebinthus*, grafting in vitro scions onto ex vitro germinated seedlings reduce production time [107]. Taking into account the advantages of in vitro over in vivo grafting, most relevant findings included in Table 1 are further discussed below.

### 2.1. Fruit Trees

#### 2.1.1. Subtropical Genera

##### Citrus

Huang et al. [53] obtained progressive restoration of rooting competence and vigor following successive micrografting of 2 mm long adult scions from *Citrus reticulata* Blanco (Ponkan mandarin orange) and *C. sinensis* (Liu Tseng sweet orange), onto 2-week-old Troyer citrange seedlings used as rootstocks. Two cm long shoots were excised from micrografts and their morphogenic competence was assessed at various levels, ca. 80% of shoots from the first micrograft developed chlorotic leaves with 50% abscission; however, after the fifth graft, all shoots showed excellent leaf development. Rooting capacity progressively increased with grafting, reaching values of 45%, 50%, and 69% for the fifth, sixth, and seventh micrografts vs. the 100% shown by juvenile material. Callus cultures initiated from grafted shoots did not form either adventitious shoots or somatic embryos. Persistence of the rooting capacity on grafted shoots led these authors to conclude that reversion of adult shoot apices to more juvenile forms had been accomplished.

##### Persea

In avocado, Pliego-Alfaro and Murashige [55] restored rooting competence in adult Duke-7 rootstock by micrografting lateral buds isolated from mature trees onto in vitro germinated seedlings. Approximately 50% of the micrografted scions regained rooting capacity although rooting percentage and number of roots per shoot (1.9) were much lower than that of juvenile material (100% rooting with 3.5 roots per shoot; adult shoots failed to root). Successive grafting did not improve either rooting or proliferation. Auxin sensitivity was similar in adult and grafted adult materials, i.e., in the presence of auxin these shoots showed leaf abscission. Grafted material grew more rapidly than their adult counterparts although it could not be maintained through successive subculturing.

Adult shoots of the Gvar-am-13 avocado rootstock showed a rooting frequency of 5%; while this increased to 35, 45, and 56% after 1, 4, and 13 micrografts respectively, in comparison to 84% for juvenile material derived from in vitro germinated seedlings. During proliferation, the behavior of micrografted shoots was similar to adult material, showing apical necrosis and low multiplication rate [56].

#### 2.1.2. Temperate Genera

##### Castanea

Fernández-Lorenzo and Fernández-López [58] evaluated the efficiency of in vitro grafting for rejuvenating *Castanea sativa* Mill. using 2 cm long scions from mature trees grafted onto in vitro-rooted juvenile shoots. Increased multiplication rate of micrografted material (2.1) was observed compared with nongrafted shoots (1.3), although no differences in rooting competence could be found between shoots of either origin (ca. 50%). Grafting up to three times did not increase rooting competence, although the multiplication rate was improved and maintained through 12 successive subcultures. More recently, Crecente-Campo et al. [59] used in vitro-derived adult material from the crown of an 80-year-old tree, for grafting onto in vitro rooted shoots derived from basal sprouts of the same tree. After five successive micrografts and subsequent in vitro subculturing, multiplication, and rooting rates of adult scions from the crown and from basal sprouts were similar.

##### Olea

Revilla et al. [62] reported successful apical micrografting of adult olive (*Olea europaea* L.) shoots of ‘Arbequina’ onto juvenile cuttings derived from germinated embryos. Improved rooting from 2% (adult) to 50% was observed in the grafted shoots. A second graft did not improve rooting in shoots derived from these grafts; however, cuttings from this material that had been transferred to a greenhouse rooted at the same rate as juvenile material (100%). Different results were obtained by Vidoy-Mercado et al. [64] with the same cultivar: increased rooting percentage from 13% (first micrograft) to 61% (fifth micrograft). Cuttings obtained from donor plants of the fifth micrograft showed 92% rooting. SSR analysis of grafted material indicated that no variation had occurred on shoots derived from the grafts.

A protocol for micrografting and micropropagation of the Iranian olive ‘Zard’ was reported by Farahani et al. [63]. Ten to fifteen mm long adult scions were grafted in vitro onto three-week-old seedlings. After micrograft establishment, shoot elongation improved with successive micrografts (3.7 cm; 4.4 cm and 5.2 cm for the 1st, 2nd and 3rd, respectively). Shoots excised from the grafts could be maintained through successive subculturing on a cytokinin-supplemented medium [63].

### 2.2. Forest Trees

#### 2.2.1. Angiosperms

##### Cedrela

The Spanish red cedar (*Cedrela odorata* L.) is an important timber tree. Robert et al. [67] collected material from >30-year-old adult trees and grafted them on greenhouse-grown seedlings; after 6 months, these plants were used as a source for scions for in vitro grafting onto juvenile rootstocks. Following 2 rounds of successive grafting, shoots could be rooted and, 6 months after acclimatization, their root appearance and plant height were similar to seedling controls.

##### Hevea

Rejuvenation effects of micrografting in *Hevea brasiliensis* (Willd.) Muell.-Arg. are highly genotype-dependent, e.g., Perrin et al. [70] obtained 60% rooting of PB235 clone after a single graft while up to six consecutive grafts were needed for clone GT1 to achieve 35% rooting. These authors used zeatin levels as a rejuvenation marker, e.g., much higher zeatin levels were found in the grafted material with improved rooting capacity than in control nongrafted shoots.

#### 2.2.2. Gymnosperms

##### Pinus

Fraga et al. [75] grafted adult >30-year-old *Pinus radiata* D. Don. buds from different clones onto juvenile cuttings, thereby demonstrating that scion age was critical for the proliferation of shoots derived from the grafts, e.g., younger scions had higher proliferation rates. Moreover, 50% of scions from basal branches sprouted vs. only 10% from apical branches. Material derived from the grafts could be established in vitro showing, after 6 months, a growth rate similar to juvenile material.

Measurements of endogenous ABA and IAA levels in *P. radiata* following micrografting onto *P. radiata* (homografts) or *P. caribaea* (heterografts) showed increased ABA linked to decreased IAA over time in grafted shoots, with peak values for ABA (32 µg g^−1^ FW) 120 days after micrografting while lowest IAA levels (3 µg g^−1^ FW) occurred after 60 days. These results were similar to those of juvenile buds and were independent of the rootstock [76].

##### Sequoia

Tranvan et al. [78] reported that scions obtained from a 500-year-old *Sequoia sempervirens* (D. D.) Endl. tree showed restoration of orthotropy following micrografting onto juvenile stocks. Huang et al. [79] also demonstrated that progressive rejuvenation occurred following successive grafting of adult material onto juvenile cuttings, e.g., 100% rooting was obtained after the fourth graft vs. 22% with the adult material. Moreover, shoot elongation and branching were similar to those of juvenile shoots. Alterations in protein phosphorylation patterns of adult shoots were observed after four successive micrografts, resembling juvenile shoots [80]. Increased rooting occurred in parallel with decreased esterase and peroxidase isoenzymes, both of which have been associated with the adult stage [18]. Huang et al. [16] also showed that four small mtDNA molecules are uniquely associated with juvenile and rejuvenated adult shoots. The mtDNA did not show variation in their sequence after prolonged subculturing. Chen et al. [81] observed a higher level of miR156 in rejuvenated shoots in comparison with adult material while the opposite was observed for miR172. sRNAs target genes involved in photorespiration and jasmonic acid (JA) mediated restoration of rooting competence showed similar expression levels in juvenile and rejuvenated adult materials. A high degree of similarity was found for epigenetic processes (chromatin remodeling and histone acetylation).

## 3. Rootstock Scion Interactions and Signaling

Progress in understanding of long distance signaling in plants and improved knowledge of communication mechanisms across the graft union in vegetable [108,109,110,111], fruit crops [111,112,113], and model species such as *Arabidopsis thaliana* [114,115], could be helpful for explaining the influence of the root system on the scion. Signaling through grafting occurs in both directions via the vascular system and it could involve different plant hormones, primary and secondary metabolites, peptides, small organic molecules, and nucleic acids, as well as water and nutrients [109,116]. Wounding can have drastic effects on cell division and regulation of morphogenesis. It triggers calcium influx into cells and ROS increases, thereby activating signaling cascades [117]. von Aderkas and Bonga [118] indicated that temporary stress caused by wounding in grafts could be responsible for partial degradation of cytoplasm of shoot meristem cells, as observed in starvation-induced stress, thereby enhancing rejuvenation; however, the in vitro stress response can vary among genotypes due to differences in physiological state and hormonal balance at the time of explanting [119].

Of particular interest for in vitro studies is the graft union position, since it has been shown to affect the nature of signals coming from the rootstock [120]. In woody perennials, grafting of lateral buds or shoot tips into the epicotyl, the stem [52,53,54,55,62,67,73,75,76,79], or the hypocotyl [57,63,64] as well as side grafting into the epicotyl or hypocotyl [51] have been described. Noticeable differences in grafting success have been found in cases where different grafting procedures have been assayed, with side-grafting being superior to top-grafting in some cases [51,74], but not in others [92].

Factors deserving consideration in rootstock scion interactions include scion size and serial grafting. Restoration of morphogenic competence have been achieved with scions of different size (Table 1); however, in vitro culture of shoot apices of maize with 1–2 leaf primordia resulted in plants that flowered at the same time as seedlings [121], while apices with 3–4 leaves only showed partial rejuvenation [122]. In *Sequoiadendrum giganteum* rejuvenated plants were obtained following meristem culture, with physiological stage of the explant being critical for success [123]. However, with *Sequoia sempervirens* micrografts, Huang et al. [79] used 1.5 cm scions and extent of rejuvenation increased with the number of grafts. With *Citrus*, Huang et al. [53] using mature scions >2 mm long and Navarro et al. [124,125] with 0.14–0.18 mm long scions (when attempting to get virus-free scions) did not observe rejuvenation on the adult material after one graft; however, phase reversal was observed after several grafts [53].

### 3.1. Signaling through Graft Union

#### 3.1.1. Mineral Nutrients and Hormones

Gregory et al. [126] indicated that control of scion growth by the rootstock is influenced by hydraulic signaling and plant hormones together with other chemical factors. Genotype of the rootstock has a crucial role and very different responses could be obtained for a given scion grafted onto different rootstocks [109]. Availability of water and nutrients are important and studies involving *Malus* have shown that a high xylem/phloem ratio in the rootstock favors increased vigor [126], while Santarosa et al. [127] reported a positive correlation between xylem area, vessel diameter, and vigor with the grapevine. According to Savvas et al. [128] and He et al. [129], the rootstock could strongly affect the nutrient status of the scion, either through inhibition of heavy metal and micronutrient uptake or by enhancing absorbance of macronutrients. However, Else et al. [130] did not detect differences in ion uptake among apple rootstocks differing in dwarfing traits. Scions could also modulate rootstock responses under conditions of limited nutrient (Pi) supply [131]. Enhanced nutrient uptake by the rootstock could be related to size of the root system, although cultural conditions might also be important, e.g., tomato plants showed strong variations in growth rate when grafted onto different rootstocks under standard cultural conditions while these differences were not noticeable when grafted plants were grown under soilless optimal growth conditions [132]. This observation should be considered when evaluating the improved growth of adult scions following graftage onto different juvenile rootstocks in vitro. As shown in Table 1 and Table 2, mineral formulations with different ionic content have been used in the vitro grafting assays, generally varying with the species in question and selecting those that induced a better growth of the scion, e.g., MS at full [52,53,55] or half strength [54,61,68]; WPM [58,59] and DKW [62] et al.

Plant hormone levels greatly affect the scion response [133]. Generally, the observed scion vigor increase has been associated with cytokinin supply from the roots, e.g., more vigorous rootstocks show a higher content of cytokinins in the xylem sap [134,135], although this has not always been the case [136]. A higher cytokinin content in the shoot would result in a more active functioning as a sink, favoring the accumulation in the shoot of mineral elements and amino acids [137,138]. Cytokinin biosynthesis in the roots is modulated by nitrate availability [139]. This ionic signal when moving through xylem and reaching the shoot could possibly interfere with auxin supply to the root decreasing root branching [140]. Intensity of polar auxin transport has been shown to be much lower in dwarfing than in semi-invigorating apple rootstocks [130,141] and similar observations regarding auxin signaling have been reported in Citrus [142].

Bud growth regulation should be considered with respect to strigolactones (SL), e.g., auxin transported basipetally throughout the stem has a positive effect on SL biosynthesis in the roots. SL moving up through the xylem would interfere with polar transport leading to bud competition to release auxin to the stem, hence negatively affecting auxin biosynthesis. CKs and SLs moving up through the xylem would have opposite effects on bud growth through interaction with BCR1 transcription factor [143]. Investigations in rice have shown that SL suppress expression of type A-ARR (Arabidopsis Response Regulators involved in cytokinin action) in the bud [144,145]. Moreover, expression levels of SL biosynthetic genes are suppressed by CKs [145], which in turn are also involved in controlling polarization of auxin transport by modifying the direction of auxin flow [146]. In tomato, transgenic lines with reduced levels of SLs showed more enhanced branching and profuse development of adventitious roots [147]. The effect of SL appears to be linked to nutrient availability, showing a stronger effect on bud growth inhibition under Pi deficiency [148]. In grafted grapevine, rootstocks producing higher levels of SL-like compounds induced scion growth reduction, and this effect is greater under N-limiting conditions [149]. Hence, this carotenoid derived hormone is important for root-shoot signaling although its role in phase change remains to be elucidated.

The inactive gibberellins in the xylem should be converted to active forms in the shoot [120]. Regnault et al. [150], using micrografted Arabidopsis plants, identified the gibberellin precursor GA_12_ as the primary mobile long distance signal in xylem sap while previous observations of Lavender et al. [151], with Douglas fir, indicated that gibberellins from the roots are responsible for initiating shoot growth in the spring. A lower xylem content of the inactive gibberellin GA_19_, at the beginning of spring, has been associated with dwarfing caused by M.9 apple rootstock; the inactive form would subsequently be converted to the active GA_1_ in the scion [136]. In mandarin grafted onto different *Citrus* rootstocks, GA biosynthesis was positively correlated with vigor [142].

The role of ABA signaling has been mainly studied in relation to stress situations [109,116,152]. The presence of higher ABA in the xylem sap is correlated with shoot growth inhibition [153,154]. With apple, greater ABA levels have been detected in scions grafted onto dwarfing rootstocks [130]. Hence, the GAs/ABA ratio in juvenile root systems is an important factor for reinvigoration of adult scions.

Jasmonic acid is mainly synthesized in leaves and flowers [116]; however, roots could also be a JA source [155,156]. In *Arabidopsis* JA is involved with upregulation of auxin biosynthesis [157], enhancing de novo root formation. The hormone precursor cis-12-oxo-phytodienoic acid moves from wounded shoots grafted onto undamaged roots where they are converted to JA and signaling pathways are activated [158]. It is not known how JA from roots might affect shoot growth [116] although there is evidence that it interacts with ABA biosynthesis in these organs [156].

Brassinosteroids are involved in root architecture [159]; however, they show a localized mode of action and do not seem to have a direct effect on long distance signaling although they are important for auxin transport [160]. Inhibition of brassinosteroids biosynthesis in apple by overexpression of MdWRKY9 induces a strong dwarfing phenotype [161]. Prassinos et al. [162] identified a number of differentially expressed genes in cherries that are mostly involved in flavonoid metabolism, brassinosteroid signaling and cell wall biosynthesis, that could be related to the earlier cessation of terminal growth and subsequent decreased size caused by dwarfing rootstocks. Warschefsky et al. [113] suggested that reduction of vigor could be induced through different independent molecular pathways.

Generally, culture media for growing in vitro grafts (Table 1 and Table 2) either lack any hormones [52,53,54,79] or have included a cytokinin [55,56,59] or a cytokinin-auxin supplement [62,75,103]. Use of either cytokinins [163,164] or gibberellins [165,166], has been associated with reversion to more juvenile forms. Based on these observations, Huang et al. [79] tried unsuccessfully to duplicate grafting effects through preincubation of adult or one-grafted *Sequoia semprevirens* shoots on media containing either benzyladenine or gibberellic acid followed by transfer to rooting medium. Experiments to study the effects of plant hormones together with grafting on rejuvenation have not been performed.

#### 3.1.2. Metabolites

A vast range of metabolites other than ions and hormones are present in xylem sap. Albacete et al. [138] identified ca. 800 primary (amino acids, sugars, sugar phosphates, organic acids, fatty acids, and polyols) and secondary metabolites (alkaloids, flavonoids, glucosinolates, and others) whose biological functions are largely unknown, while for others, e.g., sugars, it is known they play a role in signaling, e.g., trehalose-6-phospate is involved in regulating the pathway linking ontogenetic age and capacity for flowering [3,33,167]. In grapevine, the nature of these metabolites is greatly affected by rootstock [168]. Tietel et al. [169] found six out of 14 primary metabolites in phloem sap of *Citrus* scion being affected by the rootstock, whereas 42 were dependent on the rootstock–scion interaction. To identify and characterize metabolites occurring at specific developmental stages, Venema et al. [116] proposed that metabolite profiling and multivariate data mining could be useful tools.

#### 3.1.3. Proteins

Different omics studies have demonstrated that macromolecules are important as long-distance signals moving through vascular systems [170]. The FT protein, responsible for flower induction [171], has been shown to move across the graft union and accelerate reproductive development when scions of some species have been grafted onto transgenic rootstocks [172]. In addition, other proteins, i.e., cyclophilin SICyp1, affecting auxin signaling and modulating root growth [173] and RNA-phloem transport proteins, found in the phloem of grafted vegetables [110], indicate the relevance of sieve tube elements in stock-scion communication. A clear example of movement from stock to scion can be found with polygalacturonase inhibiting proteins, that enhance tolerance of pathogens in grape and tomato [174]. Other proteins possibly involved in rootstock–scion interaction are peroxidases (ROS scavenging) and Calcineurin-B-Like proteins (calcium signaling) [175]. Toscano-Morales et al. [176] demonstrated movement of the *Arabidopsis thaliana* Translationally Controlled Tumor Protein 2 (*At*TVTP2) in grafted tobacco from rootstock to scion and vice versa. Long distance movement of the protein was required for adventitious rooting. Protein concentration is important for appropriate binding to receptors and transpiration amplifies the signal; afterwards secondary signals are sent back to the whole plant via phloem [177]. In vitro cultures have low transpiration rates, and the role of protein signaling under these conditions would require further study.

#### 3.1.4. RNAs

In addition to proteins, movement of mRNAs and small RNAs from source to sink and across the graft union have also been reported [178,179,180]. mRNA-protein complexes move in the phloem across the graft union [181,182,183]. Pioneer experiments by Kudo and Harada [184] demonstrated that movement of mRNA from a tomato rootstock could alter leaf shape in a potato scion. mRNA from gibberellic acid insensitive (*gai*) gene moves from root to shoot and vice versa in micrografts of apple and pear [185,186,187]. In grapevine, mRNAs involved in stress and signaling are highly abundant [188]. Micrografts have a higher amount of these genes transmitting mRNAs than mature grafts of field-grown plants. Therefore, mRNA movement occurs in a passive or genotype and environment dependent manner. Liu et al. [189] using DsRED transgenic walnut demonstrated mRNA movement of the transgene from rootstock to wild-type scion, clearly showing the feasibility of using micrografting as a tool in fruit tree breeding as well as in physiological studies.

Short RNAs could be involved in inducing mRNA cleavage and DNA methylation in recipient cells, probably by reinforcing effects of transposons [190,191,192,193,194,195]. siRNA movement is related to conferring virus resistance from transgenic rootstocks to nontransgenic scions [196,197] or to silence endogenous genes in the scion [198]. For miRNAs, there is evidence that both miR156 [199] and miR172 [200] are graft transmissible and they have been found in phloem exudates of potato during tuber formation. They are also involved in regulating grafting effects, e.g., leaf petioles from homografts and heterografts in Citrus, showed reduced expression levels of miRNA156 in comparison to control seedlings [201]. Avocado, grafted either on juvenile (seedlings) or mature (vegetatively propagated) rootstocks, shows levels of miRNA156 and miRNA172 that are largely under scion control although transmission through graft union would be affected by the presence of leaves below it [27].

### 3.2. Changes in Gene Expression

Changes in gene expression in the scion due to rootstock-derived signals have been reported in vegetable [202] and fruit grafting [142]. In apple, changes in gene expression due to grafting on different rootstocks are related to tree size and tolerance of fire blight and other traits [203,204]. Chitarra et al. [168] reported noticeable changes in grape leaf transcript profile as affected by the rootstock, while Cookson and Ollat [205] observed changes in gene expression in the scion meristem with most affected genes being related to chromatin regulation, cell organization, and hormone signaling.

Epigenetic changes play a key role in cell reprogramming [206,207]. Although no specific studies have been carried out in woody plants, partially heritable changes in DNA methylation have been shown to occur in scions of grafted vegetables [208,209,210]. In vitro conditions enhance the occurrence of DNA methylation and histone modification processes not only during the processes of adventitious regeneration [50,211] but also during nodal culture and axillary shoot formation [212,213,214]. Changes in DNA methylation patterns are also associated with phase change, aging, and reinvigoration [10,19,20,215,216]. The close relation between hormone action and epigenetic changes is important; e.g., CKs are involved in DNA methylation [217] while recent evidence have revealed the linkage between auxin biosynthesis, transport and signaling being modulated by miRNAs and epigenetic factors, e.g., histone modification [218].

## 4. Conclusions and Future Prospects

In vitro grafting of mature trees onto juvenile rootstocks has been successfully practiced in order to reinvigorate/rejuvenate grafted scions. Optimal results have generally been obtained for scions derived from in vitro-grown shoots or greenhouse-grown plants rather than field-grown trees, notwithstanding differences between species. Higher rooting competence could be observed following sequential grafting (*Citrus*, *Garcinia*, *Persea*, and *Sequoia*, etc.) while in other genera e.g., *Castanea*, an improved proliferation rate or capacity for adventitious shoot formation (*Cedrela*) was detected. Acclimated micrografts of *Larix* and *Picea* exhibited juvenile traits and, in the case of *Larix*, explants from the scions could be successfully reintroduced in vitro. With respect to the different size of scions used, the importance of sequential grafting and the fact that phase reversal has been achieved either in the presence or absence of plant hormones, experiments are needed to study the importance of possible interactions between these factors in achieving rejuvenation. Use of molecular markers such as the relative increase of miRNA156 levels are essential to quantify the degree of reversion achieved.

Marguerit et al. [219] were able to identify genes in grape rootstock controlling specific traits of the scion and Ghanem et al. [220] have pointed out the advantages of using root system engineering to modify rootstock signaling and improve specific traits of the scion. Kundariya et al. [221] were able to induce epigenetic changes in Arabidopsis and tomato scions resulting in enhanced vigor, a trait that could be transmitted to the sexual progeny, and emphasized the importance of this as a breeding tool. Ellisson et al. [222] emphasized the feasibility of using CRISPR guide RNAs from rootstock transgenic lines to edit nontransgenic scions. These approaches, in combination with the well-established micrografting protocols indicated above (Table 1 and Table 2), could be used to modify physiological and/or ontogenic age in adult materials. Moreover, the feasibility of micrografting using genetically diverse rootstocks should be explored since interactions between divergent materials appear to enhance the occurrence of epigenetic changes [223] and specific signals, i.e., in Solanaceae, interspecific grafting caused extensive and heritable changes in DNA methylation [224], while in interspecific *Pyrus* micrografts, NACP mRNA coding proteins, affecting meristem development, have been found to move in both directions across the graft union [225].

It is still difficult to distinguish between changes in physiological (reinvigoration) or ontogenetic (rejuvenation) aging. Restoration of some juvenile traits should not be an indication that other mature traits do also undergo reversion [1,5]. Important challenges are to decipher key factors involved in long distance signaling causing changes in the adult meristem responsible for phase reversal. Moreover, evaluation of material obtained after rejuvenation should include a long term comparison of time course of maturation with an appropriate control (seedling plant) to accurately ascertain the degree of reversion that is achieved; i.e., in theory, complete rejuvenation should be attained in somatic embryo-derived plants; however, Martinez et al. [226] in *Quercus robur* found that shoot culture lines derived from somatic plantlets performed in vitro as shoot lines obtained from basal sprouts (considered as mature material with some juvenile traits), showing that only partial rejuvenation had been achieved. The use of temporarily reinvigorated trees as truly rejuvenated material could result in the emergence of young–old trees [10]. Rejuvenation associated morphological and physiological variations should be characterized at the molecular level. Zhang et al. [10] indicated that a challenge for the future should be obtaining rejuvenated individuals with traits similar to those of seedlings.

## Figures and Tables

**Table 1:** Woody species in which the micrografting of adult scions onto juvenile rootstocks was used to reinvigorate/rejuvenate the scion. Evaluation of changes in the scion other than increased growth was included.

Species	Scion Source	Scion Size	Mineral Formulation + Growth Regulators	Morphogenic Response	Reference
**FRUIT TREES: SUBTROPICAL**					
*Anacardium occidentale L.* (cv. AC-4)	Greenhouse grown plants, 4–5 years old	Shoot tips	MS	Rooting: 13.3% after one graft vs. 0% for ungrafted adult shoots	[51]
*Annona cherimola* M. (cvs. Fino de Jete, Bonita, Pazicas)	Actively growing shoots collected in spring from mature plants growing in a glasshouse	Nodal segment (1–2 cm) with a lateral bud	MS	Rooting for ‘Fino de Jete’, ‘Bonita’ and ‘Pazicas’ after 3 micrografts: 70, 60, and 50%, respectively Rooting capacity was lost when shoots were removed from the grafts, cultured on multiplication medium and subsequently rooted	[52]
*Citrus reticulata* B. (cv. Ponkan mandarin) and *Citrus sinensis* O. (cv. Liu Tseng, sweet orange)	Mature trees	Shoot tips (2 mm)	Liquid MS	Both materials showed similar performance Increased rooting after 7 micrografts: 69% Vigor and elongation of the shoots improved with repeated grafts	[53]
*Garcinia indica* (selected genotype)	In vitro shoots from nodal sections of a 20-year-old elite tree	Shoot apices (5–10 mm)	½ MS	Rooting capacity of shoots after 5 micrografts was 75% vs. 0% for ungrafted and 100% juvenile shoots	[54]
*Persea americana* M. (cv. Duke 7)	Flowering-age plants growing in greenhouse	Lateral buds with a stem piece at the base	MS + BA	Rooting: ca 50% vs. 0% (ungrafted shoots) Rooting and vigor of shoots did not improve with successive grafts	[55]
*Persea americana* M. (cv. Gvar-Am13)	Mature plants grafted in the greenhouse	Lateral buds with a stem piece at the base	N_45_K macroelements + MS microelements + BA	Rooting after 13 micrografts was 56% vs. 5% and 84% for adult and juvenile shoots, respectively Proliferation rate of micrografted and adult shoots was very poor	[56]
*Ziziphus mauritiana* L. (cv. Gola)	Grafted plants growing in a greenhouse and nodal explants cultured in vitro	Apical or axillary bud (5–10 mm)	Liquid ½ MS	Rooting of microcuttings obtained after 1 and 3 micrografts were 4 and 40% respectively, vs. 71 for juvenile material	[57]
**FRUIT TREES: TEMPERATE**					
*Castanea sativa* M. (cvs. Loura and Parede)	In vitro shoots maintained for 10 years from ‘forced’ branch segments of adult trees grafted for 75 years	Shoot apices (20 mm)	WPM + BA	Rooting capacity after 3 micrografts: ca 50% (similar to ungrafted controls) Multiplication rate after 3 micrografts: 2.1 vs. 1.3 (ungrafted shoots) No improved response in cv. Parede after one graft	[58]
*Castanea sativa* M. (clone P2)	In vitro shoots maintained for 28 years from the crown of an 80 year-old tree	Nodal segments (10 mm)	WPM + BA	After 5 micrografts and subsequent in vitro subculturing, multiplication and rooting rates were similar to material from basal sprouts of the same tree	[59]
*Juglans regia* L. (cv. Serr)	Mature trees	-	DKW + BA + IBA	After 2 micrografts, rooting capacity did not increase significantly; however, successive subculturing improved rooting	[60]
*Malus domestica* B. (cvs. Remo, Rewena, Reanda, and JTE-F rootstock)	Field-grown adult plants of 3 cultivars and JTE-F rootstock	Shoot tips	½ MS + Wuxal	The JTE-F rootstock was successfully established in vitro, while grafted material of the 3 cultivars died after 2–3 subcultures	[61]
*Olea europaea* L. (cv. Arbequina)	Mature trees (rooted cuttings growing in a greenhouse for 12 years)	Terminal shoots (10–15 mm)	DKW + BA + IBA	Rooting after 1 micrograft 57% vs. 2% for ungrafted shoots Rooting capacity did not increase after the 2nd micrograft Rooting of material from micrografts after reintroduction in vitro: 100% (same as juvenile material)	[62]
*Olea europaea* L. (cv. Zard)	Mature plants (rooted cuttings growing in a greenhouse for 4 years)	Lateral meristems (10–15 mm)	OM + Z	Shoot elongation improved with serial grafting Shoots from 3 successive grafts cultured on OM medium supplemented with 2-iP, showed a 4X proliferation rate	[63]
*Olea europaea* L. (cv. Arbequina)	Severely pruned mature tree growing in a greenhouse	Nodal segments	OM + Z	In vitro rooting increased with grafting (13% for 1st vs. 61% for 5th micrograft) Shoots derived from the grafts were rooted, acclimatized, and maintained in greenhouse for 2 years; rooting of cuttings from these plants increased with the number of grafts (92% for 5th micrograft vs. 75% for control mother plant)	[64]
**FOREST TREES: ANGIOSPERMS**					
*Acacia mangium W.*	(A) 6 month-old seedlings (juvenile) (B) 3–5 year-old seedings (mature)	Shoot apical portions (0.3–0.4 mm)	½ MS	Scions of juvenile and adult origin were micrografted successfully, although those of juvenile origin elongated faster	[65]
*Acacia mangium W.*	Lower part of the crown of 5- to 12-year-old trees	Shoot apices (0.2–0.4 mm)	½ MS	Some of the scions had composed or pinnate leaves characteristic of the juvenile stage	[66]
*Cedrela odorata* L. (8 trees selected for phenotypic quality)	Mature trees >30 years old	Shoot tips (2–4 cm)	MS	Morphogenic characters after 2 micrografts (height, internodal distance, stem phenotype, capacity for adventitious shoot formation) were similar to juvenile plants	[67]
*Faidherbia albida A.C.* (*A. albida* D.)	Suckers obtained from root fragments of a 40-year old tree grown in the greenhouse	5–10 mm stem sections with an axillary bud	½ MS	Rooting percentages and scion growth after 3 micrografts were 75% and 5.1 cm, similar to juvenile material (85% and 6.7 cm)	[68]
*Hevea brasilensis* M. A. (PB 235 and IRCA 18)	Grafted plants from clones PB 235 and IRCA 18 selected in 1950 and 1970, respectively	Shoot tips	MB + IBA + BA	Rooting capacity of 70% after 1 micrograft and 3 culture cycles vs. 3% for mature control in clone PB 235 73% rooting after 1 micrograft and 3 culture cycles vs. 7% for mature control in clone IRCA 18	[69]
*Hevea brasilensis* M. A. (clones PB 235 and GTI)	2–3-year old grafted plants from PB 235 and GTI clones selected in 1950 and 1920, respectively	Shoot tips (1–2 mm)	MB + IBA + BA	Rooting of 60% after 1 micrograft vs. 0% for mature control in clone PB 235 35% rooting after 6 micrografts vs. 0% for mature control in clone GTI Zeatin levels were higher in grafted material than in ungrafted controls	[70]
*Sterculia setigera* D.	Cuttings from 20-year-old trees grown in greenhouse for one year Nodal sections from these plants cultured in vitro for a month prior to use as microscions	Shoot apex	MS + BA	Rooting percentages were 25% (juvenile), 21% (adult) and 29% (adult after 3 micrografts) Scion vigor was similar in juvenile and grafted adult materials	[71]
**FOREST TREES: GYMNOSPERMS**					
*Larix decidua* M.	140-year-old trees	Terminal bud with removed bud scale	B	Micrografts were transferred to the greenhouse where they had plagiotropic growth	[72]
*Larix decidua* M.	140-year-old trees	Shoot tips (apical dome and first ring of leaf primordia, 0.3–0.5 mm in diameter)	Autoclaved Jiffy-7 peat pellets in sealed Petri dishes	After micrograft acclimatisation, material could be multiplied in vitro Rooting was close to 50% for grafts derived shoots while no rooting was obtained in ungrafted adult material	[73]
*Picea abies* (L.) H. K.	Rooted cuttings from an 18-year-old tree	Apical meristems 0.1–0.25 mm length	Margara macronutrients + MS micronutrients	Some grafted shoots showed active growth and juvenility	[74]
*Pinus radiata* D. D.	30-year-old grafted trees	Needle fascicle with sheath removed	1/3 QL macronutrients + MS microelements + NAA + BA	Grafted material could be established in vitro; after 6 months they showed similar growth rates as juvenile shoots	[75]
*Pinus radiata* D. D.	9-year-old trees	Apical bud (2 mm)	QL	Heterografts on *Pinus caribaea* showed better development than homografts on *Pinus radiata* ABA increase linked to IAA decrease was observed in micrografts; obtained values were similar to juvenile material	[76]
*Pseudotsuga menziesii* M.	3–4 year-old rooted cuttings from a 15-year-old tree	Apical meristems (0.1–0.25 mm)	½ QL macronutrients + MS micronutrients	Scion responses were variable: from resting buds to actively growing juvenile-like shoots	[77]
*Sequoia sempervirens* D. D. (selected tree)	In vitro stocks established from a 500-year-old tree	Shoot apices (4–5 mm)	MER	Some scions showed morphological and physiological juvenile traits After rooting and acclimatization they showed orthotropic growth	[78]
*Sequoia sempervirens* D. D.	In vitro stocks established from mature trees	1.5 cm long shoots	MS	After 4 grafts, shoots rooted at a 100% rate similar to seedlings vs. 20% rooting for grafted adult	[79]
				Phosphorylation of 32-kDa protein occurred in adult material while 31-kDa protein appeared phosphorylated in juvenile and grafted shoots	[80]
				Appearance of isoperoxidases and isoesterases in grafted and juvenile shoots	[18]
				Four small mtDNA molecules associated with juvenile and rejuvenated shoots	[16]
				Higher level of miR156 in juvenile and micrografted shoots in relation to adult material while an opposite trend was observed for miR172	[81]

B-formulation [82]; BA (6-benzyladenine); DKW (Driver and Kuniyuki, [83]); IBA (indole-3-butyric acid); MB [84]; MER (Root elongation medium, [85]); MS (Murashige and Skoog, [86]); N_45_K (MS macroelements modified as [87]); NAA (naphthalene acetic acid); OM (Olive medium, [88]); QL (Quoirin and Lepoivre, [89]); WPM (Woody Plant Medium, [90]); Z (zeatin); ZR (zeatin riboside).

**Table 2:** Woody species in which the micrografting technique was used to induce growth of the scions.

Species	Scion Source	Scion Size	Mineral Formulation + Growth Regulators	Observations	Reference
**FRUIT TREES: SUBTROPICAL**					
*Anacardium occidentale* L. (elite trees)	In vitro shoots from grafts (1–2-years old) of adult material maintained in the greenhouse	Shoot apices (6–15 mm)	Liquid MS with ½ macronutrients	Elongation of scion	[91]
**FRUIT TREES: TEMPERATE**					
*Ceratonia siliqua* L. (adult female tree)	In vitro shoots	Shoots of uniform size and diameter	MS + BA + GA_3_ + IBA	One month after grafting, growth of scions was evident	[92]
*Malus domestica* B. (cv. Royal Gala)	In vitro shoots	Shoot tips (≈30 mm)	½ MS + Wuxal	Four weeks after acclimatization of grafts, scion length reached minimum 10 cm with several leaves	[93]
*Olea europaea* L. (5 selected trees, in base to production)	I. Adult trees growing in urban zone: (a) one (ca 80-years-old) (b) four (15–20-years-old) II. Grafted plants from (a) and (b) maintained in a greenhouse for 120 days	Apical segments (with 2–3 pairs of axillary buds)	MS + 2-iP	Better shoot development and axillary shoot formation in scions from grafted plants than those derived from urban zone-grown plants	[94]
*Pistacia vera* L. (cv. Mateur)	In vitro shoots	Shoot tips (8–10 mm long) containing 2–3 axillary buds	Liquid MS	Enhanced growth of the scion and development of axillary shoots	[95]
*Pistacia vera* L. (cv. Siirt)	(a) Juvenile (1-year-old) grown in greenhouse (b) Mature trees (5–10-30-year-old) in an orchard (c) In vitro shoots from mature trees	Shoot tips (5–10 mm)	MS	Age of explant source strongly affected shoot development with poorer elongation as age increased Explants from in vitro cultures showed better performance <50% of micrografts for all age classes survived after ex vitro transplantation	[96]
*Pistacia vera* L. (cv. Siirt)	(a) Mature tree (30-year-old) in orchard (b) In vitro shoots (maintained for 1 year)	Shoot tips (4–6 mm)	MS + BA	Better growth with explants derived from in vitro cultures than with scions obtained directly from the tree	[97]
*Prunus dulcis* M. (cvs. Ferragnes and Ferraduel)	In vitro shoots	Shoots tips (4–15 mm)	MS + BA + IBA	Grafted scions showed an increase in shoot elongation and vigorous growth	[98]
*Pyrus* spp. L. (cv. Le-Cont)	In vitro shoots from field-grown mature trees	Shoot tips (meristem plus 2–3 leaf primordia) (>5 mm)	WPM + BA + IBA	Scions showed noticeable length increase, axillary shoot development, and formation of new buds 75% of micrografts survived acclimatization	[99]
**FOREST TREES: ANGIOSPERMS**					
*Acacia tortilis* (F.) H. subsp. *raddiana* (S.) B.	In vitro stocks from 2-day-old seedling and a 15-year-old tree	Shoot tips (2–8 mm)	MS	% of elongated buds was higher for juvenile scions (82%); for mature scions, a preculture step induced 42% bud elongation while for direct grafting it was 12%	[100]
*Santalum album* L. (candidate plus tree)	Materials from a 50–60 year-old field-grown tree or after in vitro establishment	Shoot tips (1–2 cm)	Liquid ½ MS	In vitro grown shoots gave better response than scions from the field	[101]
**FOREST TREES: GYMNOSPERMS**					
*Pinus pinea* L.	5 genotypes of 11-year-old trees	Needles from fascicles with sheath removed	½ WPM	Genotype effect regarding establishment and developmental rate with 43% average success	[102]
*Pinus radiata* D. D.	6-year-old trees	Apical bud excised from brachyblasts	QL + IBA + BA	Apical buds in grafts established during summer gave better response	[103]

BA (6-benzyladenine); GA_3_ (Gibberellic acid); IBA (indole-3-butyric acid); MS (Murashige and Skoog, [86]); 2-iP (2-isopentenyl adenine); QL (Quoirin and Lepoivre, [89]); WPM (Woody Plant Medium, [90]).

## Data Availability

Figures and tables on this manuscript have not been taken from other publications.
